# Inhibitory Effects of Diketopiperazines from Marine-Derived *Streptomyces puniceus* on the Isocitrate Lyase of *Candida albicans*

**DOI:** 10.3390/molecules24112111

**Published:** 2019-06-04

**Authors:** Heegyu Kim, Ji-Yeon Hwang, Jongheon Shin, Ki-Bong Oh

**Affiliations:** 1 Department of Agricultural Biotechnology, College of Agriculture and Life Sciences, Seoul National University, Seoul 08826, Korea; hqhqeori@snu.ac.kr; 2 Natural Products Research Institute, College of Pharmacy, Seoul National University, Seoul 08826, Korea; yahyah7@snu.ac.kr

**Keywords:** diketopiperazine, marine actinomycete, *Streptomyces puniceus*, *Candida albicans*, isocitrate lyase

## Abstract

The glyoxylate cycle is a sequence of anaplerotic reactions catalyzed by the key enzymes isocitrate lyase (ICL) and malate synthase, and plays an important role in the pathogenesis of microorganisms during infection. An *icl*-deletion mutant of *Candida albicans* exhibited reduced virulence in mice compared with the wild type. Five diketopiperazines, which are small and stable cyclic peptides, isolated from the marine-derived *Streptomyces puniceus* Act1085, were evaluated for their inhibitory effects on *C. albicans* ICL. The structures of these compounds were elucidated based on spectroscopic data and comparisons with previously reported data. Cyclo(L-Phe-L-Val) was identified as a potent ICL inhibitor, with a half maximal inhibitory concentration of 27 μg/mL. Based on the growth phenotype of the *icl*-deletion mutants and *icl* expression analyses, we demonstrated that cyclo(L-Phe-L-Val) inhibits the gene transcription of ICL in *C. albicans* under C_2_-carbon-utilizing conditions.

## 1. Introduction

The glyoxylate cycle exists in a wide range of organisms, such as archaea, bacteria, fungi, plants, and nematodes. This cycle plays roles in cell metabolic processes as an anaplerotic pathway of the tricarboxylic acid cycle catalyzed by isocitrate lyase (ICL) and malate synthase, which is a key component of the glyoxylate cycle [1]. In microorganisms this cycle is essential for the uptake and utilization of non-fermentable carbon sources, such as ethanol, acetate, and fatty acids [2]. During host infection, pathogenic microorganisms including *Mycobacterium tuberculosis* and *C. albicans* cause up-regulation of the glyoxylate cycle [3,4]. In particular, the *icl* gene of *Candida albicans* is strongly activated when cells are exposed to macrophages. In mice, an *icl*-deletion mutant of *C. albicans* was unable to utilize C_2_ carbon substrates and had diminished virulence compared with the wild-type strain [2,5,6,7,8]. As this cycle does not exist in mammalian cells, ICL appears to be a prospective target for the development of antifungal drugs. Numerous studies have been conducted to find a potent ICL inhibitor [9,10,11].

Diketopiperazines are simple cyclic peptides composed of two amino acids with conformationally constrained heterocyclic structures, which are stable to proteolysis [12]. These compounds are a relatively unexplored class of bioactive peptides. The naturally occurring bioactive diketopiperazines are produced by a variety of organisms, such as bacteria (*Bacillus* sp. and *Streptomyces* sp.), fungi (*Alternaria alternata* and *Penicillium* sp.), and marine sponges (e.g., *Dysidea fragilis*) [13]. Recently, the interest in these compounds has increased due to their various characteristics. Diketopiperazines possess a number of diverse biological properties, such as antimicrobial activity [12], antitumor activity [14], antiviral activity [15], inhibition of quorum-sensing signaling [16], plant-growth promotion [17], inhibition of carbonic anhydrases [18], and inhibition of aflatoxin production [19].

As part of our ongoing search for inhibitors of *C. albicans* ICL, we encountered *Streptomyces puniceus* Act1085, isolated from marine sediment from Jeju Island, Republic of Korea [20]. In this study, an organic extract of the culture broth demonstrated inhibitory activity toward *C. albicans* ICL. The subsequent activity-guided separation of the organic extract led to the isolation of five diketopiperazines. Although these diketopiperazines have been reported to possess biological properties, such as antimicrobial, antitumor, anti-inflammatory, and neuroprotective activities [21,22,23], the inhibitory activity of the isolated compounds toward ICL has not yet been explored. Thus, we investigated the potential use of these compounds as inhibitors of *C. albicans* ICL.

## 2. Results

### 2.1. Isolation and Structural Elucidation of Diketopiperazines

**Figure 1:**
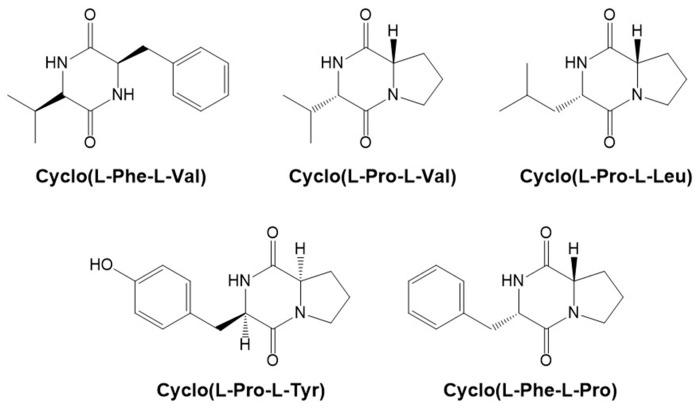
Structures of diketopiperazines isolated from *S. puniceus* Act1085.

The Act1085 strain was cultured for seven days in MTYB medium and fractionated using equal volumes of n-hexane, ethylacetate (EtOAc), and n-butanol. Based on the results of the *C. albicans* ICL activity assay, the EtOAc fraction was separated by reverse-phase C_18_ vacuum flash chromatography and preparative high-performance liquid chromatography (HPLC) to yield five compounds. Using combined spectroscopic analyses, including ^1^H, ^13^C nuclear magnetic resonance (NMR), 2D NMR spectral analyses (COSY, HMQC, and HMBC), and UV data, the isolated compounds were identified as diketopiperazines: cyclo(L-Phe-L-Pro) [24], cyclo(L-Pro-L-Leu) [25], cyclo(L-Pro-L-Tyr) [24], cyclo(L-Pro-L-Val) [25], and cyclo(L-Phe-L-Val) [26] (Figure 1). The spectroscopic data obtained from the isolated compounds are consistent with previously reported values.

### 2.2. ICL Inhibitory Activity and Antifungal Activity of Diketopiperazines

**Figure 2:**
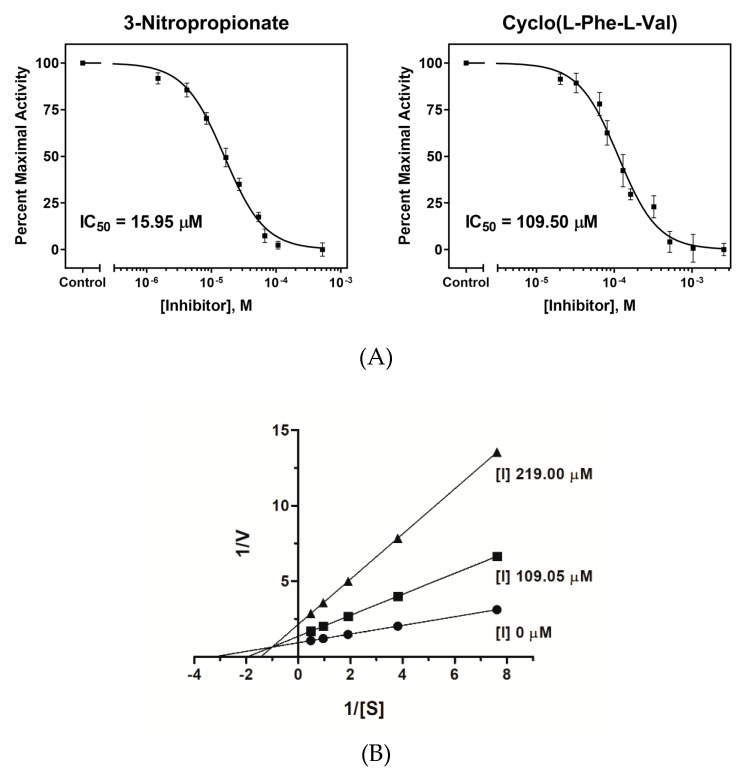
(**A**) Comparison of the dose-dependent curves of the cyclo(L-Phe-L-Val) (test compound) and 3-nitropropionate (positive control) inhibitory activity toward the ICL enzyme from *C. albicans* ATCC10231. The data were analyzed using non-linear regression curve fitting in GraphPad software ver. 8.0 (Prism). The vertical bars indicate the standard errors (*n* = 3). (**B**) Lineweaver-Burk plot for ICL inhibition in the presence of cyclo(L-Phe-L-Val). S and V represent the substrate concentration (mM) and reaction velocity (ΔA_324nm_/min), respectively. Each data point represents the mean of three experiments.

Isolated diketopiperazines were tested for ICL inhibitory activity according to methods reported previously [27]. The inhibitory concentrations (IC_50_) values of the isolated compounds are shown in Table 1. Of the isolated diketopiperazines, cyclo(L-Pro-L-Leu) and cyclo(L-Pro-L-Val) demonstrated weak inhibitory activity toward the ICL enzyme, with IC_50_ values of 533.79 μM and 516.28 μM, respectively (Table 1). Cyclo(L-Phe-L-Val) exhibited the strongest inhibitory activity of the test compounds but demonstrated inhibitory potency that was less than that of 3-nitropropionate, with IC_50_ values of 109.50 μM and 15.95 μM, respectively (Figure 2a). The others exhibited no inhibitory activity. To determine the type of inhibition, kinetic analysis was performed with cyclo(L-Phe-L-Val) (inhibitor) and phenylhydrazine (substrate). The inhibitor constant (Ki) was calculated from the Dixon plot. The results showed that cyclo(L-Phe-L-Val) behaved as a mixed inhibitor, with a K_i_ value of 64.86 μM (Figure 2b). Fungal growth inhibition tests indicated that diketopiperazines at a concentration of 256 μg/mL did not exhibit inhibitory effects on SC5314 cultured in glucose (Table 1).

### 2.3. Inhibition of C_2_ Substrate Utilization

The glyoxylate cycle is necessary for virulence in *C. albicans*, which can survive in macrophages under abundant carbon sources, such as fatty acids or their breakdown products [5,8]. When *C. albicans* is phagocytosed by a macrophage, the shift in metabolism from glycolysis to the glyoxylate cycle is activated so that the cells can utilize C_2_ carbon sources. It was expected that ICL inhibitors would reduce the nutrient uptake capacity and impede survival of the pathogen in the macrophage. To determine whether cyclo(L-Phe-L-Val) affects C_2_ substrate use, *C. albicans* strains SC5314, ATCC10231, ATCC10259, ATCC11006, and ATCC18804 were grown in YNB liquid broth containing either 2% glucose or 2% acetate as the sole carbon source. Cyclo(L-Phe-L-Val) exhibited a potent inhibitory effect on *C. albicans* in acetate (minimum inhibitory concentration of 32–64 μg/mL) but no inhibitory effect on *C. albicans* in glucose (Table 2). These results demonstrate that cyclo(L-Phe-L-Val) affects ICL-mediated proliferation of the fungus under C_2_-carbon-utilizing conditions.

### 2.4. Effects of Cyclo(L-Phe-L-Val) on Growth Phenotype and icl Expression

**Figure 3:**
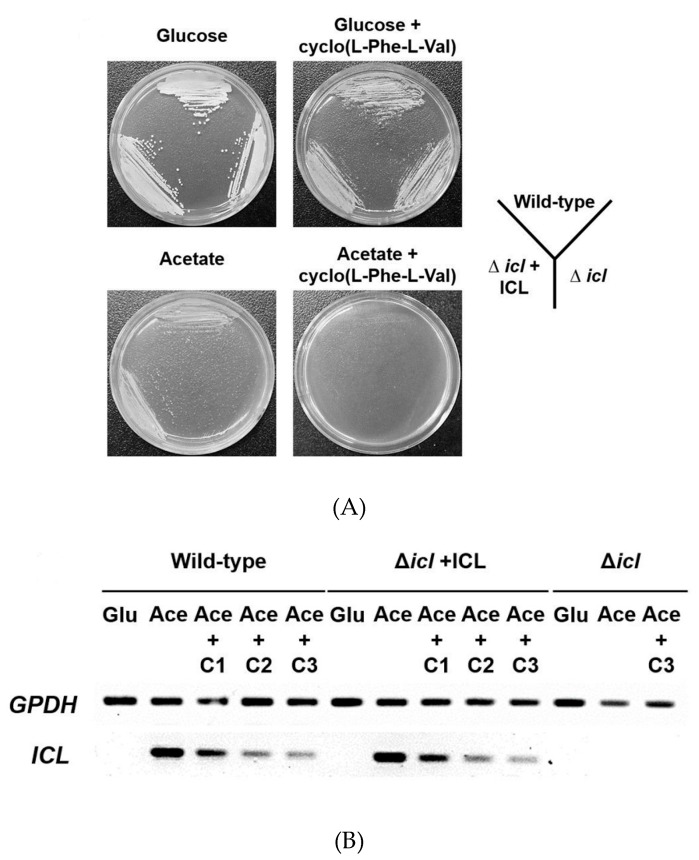
Analysis of growth phenotypes and *icl* mRNA expression. (**A**) *C. albicans* SC5314 (wild-type), MRC10 (Δ*icl*), and MRC11 (Δ*icl* + ICL) were cultured on YNB agar plates containing the indicated carbon source (2% glucose or 2% potassium acetate) with or without 32 μg/mL cyclo(L-Phe-L-Val) for 2 days at 28 °C. (**B**) Strains were grown until the mid-log phase in minimal YNB liquid medium containing 2% glucose. Cells were collected by centrifugation and transferred to the same medium containing 2% glucose (Glu), 2% potassium acetate (Ace), or 2% potassium acetate (Ace) plus cyclo(L-Phe-L-Val) (C1: 8 μg/mL; C2: 16 μg/mL; and C3: 32 μg/mL) and cultured for 4 h at 28 °C. Total RNA was prepared from these cells, and *icl* mRNA expression was analyzed by semi-quantitative RT-PCR. The *GPDH* housekeeping gene was evaluated as a loading control.

To determine whether the cell phenotype of the *icl*-deletion mutant is affected by the presence of cyclo(L-Phe-L-Val), a growth assay was conducted using *C. albicans* SC5314 (wild type) and two *icl*-deletion mutants (MRC10 and MRC11). After pre-culture, these strains were streaked onto YNB agar containing 2% glucose or 2% potassium acetate with or without 32 μg/mL cyclo(L-Phe-L-Val). All strains grew normally on both the plates with glucose and with glucose plus the compound. However, MRC10 did not grow when acetate was the sole carbon source. Furthermore, none of the tested strains exhibited growth on the YNB agar plate with cyclo(L-Phe-L-Val) (Figure 3a). We further conducted semi-quantitative reverse-transcription (RT)-PCR to assess the effects of cyclo(L-Phe-L-Val) on ICL expression. No ICL-specific PCR product was detected in the cultures when SC5314 and MRC11 were grown in YNB liquid broth containing glucose. However, *icl* was strongly induced when these cells were cultured in YNB broth containing acetate. The intensity of the PCR band corresponding to the *icl* product decreased with increasing cyclo(L-Phe-L-Val) concentrations in the cells grown under *icl* expression conditions (Figure 3b). *GPDH* expression was detected in all treatments regardless of cyclo(L-Phe-L-Val) exposure. These results indicate that cyclo(L-Phe-L-Val) inhibits *icl* expression in *C. albicans* under C_2_-carbon-utilizing conditions.

## 3. Discussion

The glyoxylate cycle, which is an anaplerotic pathway of the tricarboxylic acid cycle, is well documented in prokaryotes and eukaryotes. Diverse pathogenic fungi utilize the glyoxylate cycle during host infection. The function of this cycle has been confirmed by the analysis of mutant pathogenic microorganisms deficient in ICL and malate synthase, which are key components of the glyoxylate cycle. Research on candidiasis in mice has revealed that *C. albicans*, the most serious pathogenic fungus in humans, requires ICL to be fully virulent. As the glyoxylate cycle does not exist in mammalian cells, ICL is a promising target for antimicrobial agents. Therefore, we are interested in identifying an ICL inhibitor derived from natural products. In this study, five diketopiperazine compounds were isolated from the marine-derived *S. puniceus* Act1085, and their structures and inhibitory activities toward the *C. albicans* ICL enzyme were evaluated. Our work concluded that cyclo(L-Phe-L-Val) possesses ICL inhibitory activity. Analyses of the phenotype of *icl*-deletion mutants by growth assays and semi-quantitative RT-PCR demonstrated that this compound inhibits *ICL* expression in *C. albicans* under C_2_-carbon-utilizing conditions.

Diketopiperazines possess various biological properties [12,14,15,16,17,18,19]. Many research groups have focused on the pharmacological potential of diketopiperazines due to their advantages in medical chemistry, such as stability against proteolysis, mimicry of peptidic pharmacophoric groups, conformational rigidity, substituent group stereochemistry, and the existence of donor and acceptor groups for hydrogen bonding (which favor interactions with targets) [28]. Furthermore, diketopiperazines can be easily synthesized and isolated from natural products. In this study, five diketopiperazines, cyclo(L-Phe-L-Pro), cyclo(L-Pro-L-Leu), cyclo(L-Pro-L-Tyr), cyclo(L-Pro-L-Val), and cyclo(L-Phe-L-Val), were obtained from a marine actinomycete, *S. puniceus*, using activity-guided separation processes. Among these diketopiperazines, cyclo(L-Pro-L-Leu), cyclo(L-Pro-L-Val), and cyclo(L-Phe-L-Val) exhibited ICL inhibitory activity, with IC_50_ values in the range of 27–112 μg/mL (Table 1). The structures of the isolated diketopiperazines exhibiting ICL inhibition activity contained an isopropyl moiety, which suggests that the isopropyl moiety plays a role in the inhibition of ICL activity.

In *M. tuberculosis*, a bacterium, and *C. albicans*, a fungus, virulence led to expression of *icl*, which encodes a component of the glyoxylate cycle during persistent infection of macrophages [5,8]. These findings indicate that inhibitors of the glyoxylate cycle may reduce nutrient uptake capacity, leading to the death of these pathogens in macrophages. Thus, the effect of cyclo(L-Phe-L-Val) on *C. albicans* ICL was investigated by analyzing the growth phenotype and *icl* transcript levels in *C. albicans* SC5314 (wild-type) and *icl*-deletion mutants (MRC10 and MRC11) [8]. The growth assay revealed that cyclo(L-Phe-L-Val) specifically inhibits the ICL enzyme, because no growth of SC5314 or MRC11 was observed on the YNB agar plates containing acetate plus 32 μg/mL of the compound. Moreover, *icl* transcript levels declined as a result of treatment with cyclo(L-Phe-L-Val). Overall, this research uncovered a compound of the diketopiperazine class as a promising antifungal agent that acts by suppressing *C. albicans* pathogenicity.

## 4. Materials and Methods

### 4.1. General Experimental Procedure

The NMR instruments and mass spectrometry equipment have been described in a previous report [29]. HPLC analysis was conducted using the Shimadzu SCL-10A (Shimadzu, Tokyo, Japan) control system connected to the RID-10A refractive index detector (Shimadzu) and UV-Vis SPD-10A detector (Shimadzu). All organic solvents were of analytical reagent grade and purchased from Fisher Scientific (Fair Lawn, NJ, USA).

### 4.2. Bacterial and Fungal Strains

*S. puniceus* Act1085 was used as the diketopiperazine-producing strain. *C. albicans* SC5314 (ATCC MYA-2876) (wild-type), MRC10 (Δ*icl*) (*icl*-deletion mutant), MRC11 (Δ*icl* + ICL) (*icl*-deletion mutant), ATCC10261, ATCC18804, and ATCC11006 were used for the growth assay. *C. albicans* ATCC10231 was used to obtain recombinant ICL. Prof. Michael C. Lorenz (University of Texas Health Science Center, Houston, TX, USA) kindly provided the ICL mutants [8].

### 4.3. Fermentation and Isolation of Diketopiperazines

*S. puniceus* Act1085 was streaked onto GTYB agar plates and incubated at 28 °C for 5 days. The composition of the GTYB medium was 2 g/L tryptone, 1 g/L yeast extract, 1 g/L beef extract, 16 g/L agar, and 10 g/L glucose. The colonies were brown in color and produced white spores. Spores of Act1085 were cultivated in 2 L flasks containing 300 mL MTYB broth for fermentation. The MTYB medium composition was 2 g/L tryptone, 1 g/L yeast extract, 1 g/L beef extract, and 10 g/L mannitol. After cultivation for 1 week under static conditions at 28 °C, the culture broth (40 L) was filtered through filter paper to remove mycelia, and the filtered culture broth was sequentially fractionated using equal volumes (80 L) of n-hexane, EtOAc, and n-butanol to obtain 146 mg of n-hexane extract, 1.7 g of EtOAc extract, and 3.9 g of n-butanol extract. Based on the results of the ICL activity assay (IC_50_ values of n-hexane, EtOAc, and n-butanol extracts were >256 μg/mL, 47 μg/mL, and >256 μg/mL, respectively), the EtOAc extract (1.7 g) was selected for further separation experiments. The EtOAc extract was subjected to reverse-phase flash column chromatography using a column packed with YMC Gel ODS-A (S-75 μm) and eluted with MeOH-H_2_O (gradient from 0:100 to 100:0), acetone, and EtOAc to produce seven fractions. Based on the bioactivity analyses, the H_2_O-MeOH (60:40, 325 mg) fraction was isolated and purified by semi-preparative reverse-phase HPLC (Agilient C_18_ column, 10.0 × 250 mm; 2 mL/min flow rate; UV 254, 365 nm detection; 40:60 H_2_O-MeOH in 50 min) to yield five diketopiperazines: cyclo(L-Phe-L-Pro) (t_R_ = 8.3 min, 7.2 mg), cyclo(L-Pro-L-Val) (t_R_ = 10.7 min, 5.2 mg), cyclo(L-Pro-L-Leu) (t_R_ = 21.3 min, 10.3 mg), cyclo(L-Pro-L-Tyr) (t_R_ = 28.5 min, 7.4 mg), and cyclo(L-Phe-L-Val) (t_R_ = 37.2 min, 6.4 mg).

### 4.4. ICL Inhibition Assay

Before evaluation of the ICL inhibitory activity of the test compounds, the recombinant ICL from *C. albicans* was prepared according to methods described in a previous report [27]. The ICL inhibitory activity of the test compounds was determined according to a previously described method [30,31]. The ICL inhibition assay is based on the principle that glyoxylate phenylhydrazone forms in the reaction reagent after treatment with isocitrate and phenylhydrazine. Each test compound was dissolved in DMSO. The reaction mixture consisted of 20 mM sodium phosphate buffer (pH 7.0), 1.27 mM threo-DL(+)isocitrate, 3.75 mM MgCl_2_, 4.1 mM phenylhydrazine, and 2.5 μg/mL purified ICL and was incubated with or without the test compounds at a concentration range of 1–128 μg/mL at 37 °C for 30 min. The increase in intensity of absorbance resulting from the formation of glyoxylate phenylhydrazone was observed using a spectrophotometer (Shimadzu) at a wavelength of 324 nm. The inhibitory activity of the test compound was calculated relative to that of the DMSO control (*n* = 3). The methods for determination of the IC_50_ value and ICL enzyme concentrations have been described in previous reports [29]. A known ICL inhibitor, 3-nitropropionate, was used as a reference control [32].

### 4.5. In Vitro Growth Assay

*C. albicans* strains were cultured in YNB broth containing 2% glucose at 28 °C for 24 h, centrifuged at 15,000× *g* for 1 min, and washed twice with sterile distilled water. Each test compound was dissolved in DMSO and diluted with YNB broth containing 2% glucose or 2% potassium acetate at concentrations ranging from 1 to 256 μg/mL. Additional DMSO was added to the medium at a final concentration of 0.5%. The fungal strain culture (20 μL) was poured into a 96-well assay plate at a final concentration of 1 × 10^4^ cells/mL and total volume of 100 μL. The culture plates were incubated at 28 °C for 3 days. The positive control was amphotericin B, which is a known antifungal compound.

### 4.6. Growth Phenotype and Icl Expression Analysis

*C. albicans* SC5314 (wild type) and *icl*-deletion mutants (MRC10 and MRC11) were cultured in YNB broth containing 2% glucose at 28 °C on a rotary shaker for 24 h. Cells were collected and washed as described above. The harvested cells were streaked on YNB agar plates containing 2% glucose, 2% potassium acetate, or 2% potassium acetate plus 32 μg/mL cyclo(L-Phe-L-Val) and incubated at 28 °C for 2 days. For *icl* expression analysis, the harvested cells were added to YNB liquid broth containing 2% glucose, 2% potassium acetate, or 2% potassium acetate plus cyclo(L-Phe-L-Val) (8, 16, or 32 μg/mL) and incubated at 28 °C for 4 h. RNA extraction, cDNA synthesis, and semi-quantitative RT-PCR were performed using previously described methods [33]. *GPDH* was used as the loading control.

## Figures and Tables

**Table 1:** Inhibitory activity of isolated diketopiperazines toward the ICL enzyme and growth of *C. albicans* SC5314. 3-Nitropropionate was used as a reference inhibitor of ICL. Amphotericin B was used as a standard antifungal drug.

Compound	ICL IC_50_, μM (μg/mL)	MIC (μg/mL)
		Glucose
Cyclo(L-Phe-L-Val)	109.50 ± 4.17 (27.74 ± 2.24)	>256
Cyclo(L-Pro-L-Val)	516.28 ± 9.18 (101.32 ± 4.22)	>256
Cyclo(L-Pro-L-Leu)	533.79 ± 3.12 (112.24 ± 1.94)	>256
Cyclo(L-Phe-L-Pro)	>1048.75 (>256)	>256
Cyclo(L-Pro-L-Tyr)	>984.16 (>256)	>256
3-Nitropropionate	15.94 ± 2.13 (1.90 ± 1.57)	>256
Amphotericin B	ND ^1^	1

^1^ ND means not determined.

**Table 2:** Effect of cyclo(L-Phe-L-Val) on *C. albicans* strains grown in glucose or acetate as sole carbon source. *C. albicans* cells (1 × 10^4^ cells/mL) were incubated with varying concentrations of cyclo(L-Phe-L-Val) for 72 h at 28 °C in YNB medium containing 2% glucose or 2% potassium acetate.

Strain	MIC (μg/mL)			
	Glucose		Acetate	
	Cyclo(L-Phe-L-Val)	Amph B ^1^	Cyclo(L-Phe-L-Val)	Amph B ^1^
SC5314	>256	1	32	0.5
ATCC10231	>256	1	32	0.5
ATCC10259	>256	1	64	0.5
ATCC11006	>256	0.5	32	0.5
ATCC18804	>256	1	64	0.5

^1^ Amphotericin B (Amph B) was used as a standard antifungal drug.
